# A weakly supervised deep learning framework for automated PD-L1 expression analysis in lung cancer

**DOI:** 10.3389/fimmu.2025.1540087

**Published:** 2025-03-31

**Authors:** Feng Jiao, Zhanxian Shang, Hongmin Lu, Peilin Chen, Shiting Chen, Jiayi Xiao, Fuchuang Zhang, Dadong Zhang, Chunxin Lv, Yuchen Han

**Affiliations:** ^1^ Department of Oncology, Renji Hospital, School of Medicine, Shanghai Jiao Tong University, Shanghai, China; ^2^ Department of Pathology, Shanghai Chest Hospital, School of Medicine, Shanghai Jiaotong University, Shanghai, China; ^3^ Department of Clinical and Translational Medicine, 3D Medicines Inc., Shanghai, China; ^4^ School of Life Science and Technology, Tongji University, Shanghai, China; ^5^ Department of Oncology, Shanghai Punan Hospital of Pudong New District, Shanghai, China

**Keywords:** PD-L1, TPS, automated scoring, MiLT, lung cancer

## Abstract

The growing application of immune checkpoint inhibitors (ICIs) in cancer immunotherapy has underscored the critical need for reliable methods to identify patient populations likely to respond to ICI treatments, particularly in lung cancer treatment. Currently, the tumor proportion score (TPS), a crucial biomarker for patient selection, relies on manual interpretation by pathologists, which often shows substantial variability and inconsistency. To address these challenges, we innovatively developed multi-instance learning for TPS (MiLT), an innovative artificial intelligence (AI)-powered tool that predicts TPS from whole slide images. Our approach leverages multiple instance learning (MIL), which significantly reduces the need for labor-intensive cell-level annotations while maintaining high accuracy. In comprehensive validation studies, MiLT demonstrated remarkable consistency with pathologist assessments (intraclass correlation coefficient = 0.960, 95% confidence interval = 0.950-0.971) and robust performance across both internal and external cohorts. This tool not only standardizes TPS evaluation but also adapts to various clinical standards and provides time-efficient predictions, potentially transforming routine pathological practice. By offering a reliable, AI-assisted solution, MiLT could significantly improve patient selection for immunotherapy and reduce inter-observer variability among pathologists. These promising results warrant further exploration in prospective clinical trials and suggest new possibilities for integrating advanced AI in pathological diagnostics. MiLT represents a significant step toward more precise and efficient cancer immunotherapy decision-making.

## 1 Introduction

In recent years, the application of immune checkpoint inhibitors (ICIs) such as programmed death ligand-1 (PD-L1) inhibitors has led to remarkable advancements in the treatment of various malignancies (1–3), demonstrating significant improvements in mortality rates for patients with melanoma (4), lung cancer (5), head and neck cancers (6), and esophageal cancer (7). However, clinical studies indicate that immunotherapy is not universally effective (8, 9). Therefore, it is crucial to identify the patients most likely to benefit from treatment with PD-L1 checkpoint inhibitors. PD-L1 has emerged as a common biomarker predicting response to immunotherapy in lung cancer (10, 11). The tumor proportion score (TPS), indicating the percentage of tumor cells that positively express PD-L1, serves as a primary indicator to identify patients who are likely to respond to ICI treatment (11–13). Clinical trials have shown that higher expression levels of PD-L1 on tumor cells correlate with improved therapeutic outcomes (14, 15), and the expression of PD-L1 also determines whether ICIs are recommended as a first-line treatment option (16). Consequently, accurate assessment of PD-L1 expression plays a critical role in clinical practice. The manual scoring of PD-L1 by different pathologists may result in inconsistent results (10). Automated image analysis could serve as a supportive tool for pathologists, aiming to reduce the variability associated with subjective human assessments and enhance overall efficiency (10).

With the continuous advancements in artificial intelligence (AI) and pathology scanning technologies, various deep learning (DL) techniques and models have been developed for analyzing pathological images, significantly broadening the scope of diagnostic pathology (17). This encompasses applications such as segmentation of tissue regions utilizing whole slide images (WSIs), detection of metastatic cancer, and classification of cancer grades (18). DL-based detection techniques for pathological images have demonstrated promising results in the detection of various cancers, including lung cancer (19), breast cancer (20), and rectal cancer (21), approaching the diagnostic accuracy level achieved by pathologists.

Most of the models developed for the quantitative analysis of pathological images utilize strong supervision learning methods (22), requiring substantial pixel-level annotated data for training, which enables the trained models to achieve high levels of accuracy (23). For instance, several teams have proposed systems that automatically predict TPS using the fully supervised learning method (24), achieving a high intra-class correlation coefficient of approximately 90%, which indicates a significant level of agreement with expert pathologists in the analysis of TPS (25).

However, this fully supervised learning method and multistep process may faces significant challenges (26). These methods require experienced pathologists to manually annotate numerous tumor regions and tumor cells for model training, which are costly and time-consuming (27). For example, to build an automated tumor proportion scoring for PD-L1 expression based on multistage ensemble strategy, Zhiyong Liang and his team constructed a cell dataset using 4264 patches of size 512 × 512 pixels, which are consisted of more than 1.5 million cells of tumor cells and normal cells (28). In addition, during annotation process, poorly trained annotators may produce low-quality annotated samples, leading to diminished model performance.

In the past few years, to address these issues, many researchers have transformed the WSI classification problem into a weakly supervised task. This approach requires only a single overall label for each WSI, eliminating the need for pixel-level annotations. Currently, multiple instance learning (MIL) has been widely utilized to tackle these weakly supervised tasks, demonstrating positive results (29). MIL is a form of supervised learning in which the learner is provided not with a set of individually labeled instances but with a collection of labeled bags, each containing numerous instances (30). Nahhas and his colleagues applied an attention-based MIL technique to predict genetic biomarkers from WSIs (31). Similarly, Farsangib and his team developed a model using MIL to diagnose acute lymphoblastic leukemia (ALL), achieving an accuracy of 96.15% (32, 33). Mustafa Umit Oner proposed a model in a pan-cancer study revealing spatial resolution of tumor purity within histopathology slides using only sample-level labels during training (34). MIL models have been successfully applied to various digital pathology tasks, and in this study we try to utilize the MIL method for predicting the TPS of PD-L1 in lung cancer.

In contrast to previous studies based on multistage ensembled supervised models and required numerous annotations of tumor regions and various cells. We proposed a TPS prediction tool multi-instance learning for TPS (MiLT), aiming to achieve accurate predictions using the MIL method, thereby reducing the time costs of annotation. This study addresses the gap in current research by providing a novel approach to TPS prediction that minimizes the need for extensive manual annotations, potentially standardizing PD-L1 evaluation and improving clinical decision-making.

## 2 Materials and methods

### 2.1 Materials

In this study, 439 samples were collected as model building and internal testing cohort, and another 104 samples were collected as external testing cohort, which came from Renji Hospital and Shanghai Chest Hospital. All the samples were processed as follows: Firstly, Samples were prepared and stained on the Dako Autostainer Link 48 platform using the PD-L1 IHC 22C3 pharmDx kit (Dako, Carpenteria, CA, USA). All slides were digitized by a KBFIO FKPro-120 slide scanner at 20 magnification (0.475 mm/pixel). All the WSIs used for in this study were evaluated by a specialist pathologist, with each WSI providing an accurate TPS value, which was then confirmed by a second pathologist to ensure reliability and accuracy in our study. All participants provided informed consent prior to sample collection, and data were anonymized to protect participant privacy. The consent process included detailed information about the purpose of the study, the procedures involved, and the potential risks and benefits of participation. Participants were assured that their participation was voluntary and that they could withdraw at any time without any consequences to their medical care. The study adhered to the principles of the Declaration of Helsinki and its amendments. Ethical approval for this study was obtained from the Renji Hospital affiliated to Shanghai Jiao Tong University, with the approval number 2023-116-C. Data handling and storage were conducted in accordance with national and institutional guidelines to ensure the confidentiality and security of participant information.

### 2.2 Overall flow chart

The workflow for the TPS prediction module is outlined as follows. The training dataset consists of immunohistochemistry (IHC) stained WSIs from nearly a thousand patients, with each image labeled at the sample level to indicate its TPS. The process begins with the evenly cropping of WSIs into 256 x 256 pixel patches. A tumor extraction module, based on a classification model, processes these images to isolate patches that contain tumor regions. The extracted patches are then randomly divided into 100 bags, which, along with the corresponding WSI labels, form the dataset for training the MIL module. Finally, the trained model is tested using both internal and external data, and the predicted TPS results are compared to the ground truth provided by pathologists. Model performance is evaluated using parameters such as intraclass correlation coefficient (ICC) and kappa statistics.

### 2.3 Tumor extraction module

Prior to data collection from the images, this project employs a convolutional neural network (CNN)-based classification model to differentiate tumor regions from non-tumor tissue regions. This ensures that the subsequent MIL model does not overfit to non-tumor regions, which can introduce noise in TPS predictions and affect overall accuracy. The whole tumor patch extraction module includes two steps: firstly, identifying tissue regions inside WSI by applying OTSU thresholding on greyscale image. Then, cropping non-overlapping 256 x 256 patches at 20 x level over tissue regions and select tumor patches using a MobileNet-V2 classification model. The classification model utilized is, pre-trained on the ImageNet dataset, with the last seven layers unfrozen for further training. Following the convolutional layers, the architecture includes a Flatten layer and a dense layer. In this project, a fully supervised approach was primarily employed for tumor detection of WSIs. The extracted tumor regions were used for the subsequent training of a model to evaluate PD-L1 expression, aimed at reducing the potential influence of unrelated background regions and normal tissues on the training model. The classification model was trained with approximately 130,000 patches, with tumor cells were manually annotated on a patch-by-patch basis to facilitate the training of the tumor segmentation model.

### 2.4 MIL module

**Figure 1:**
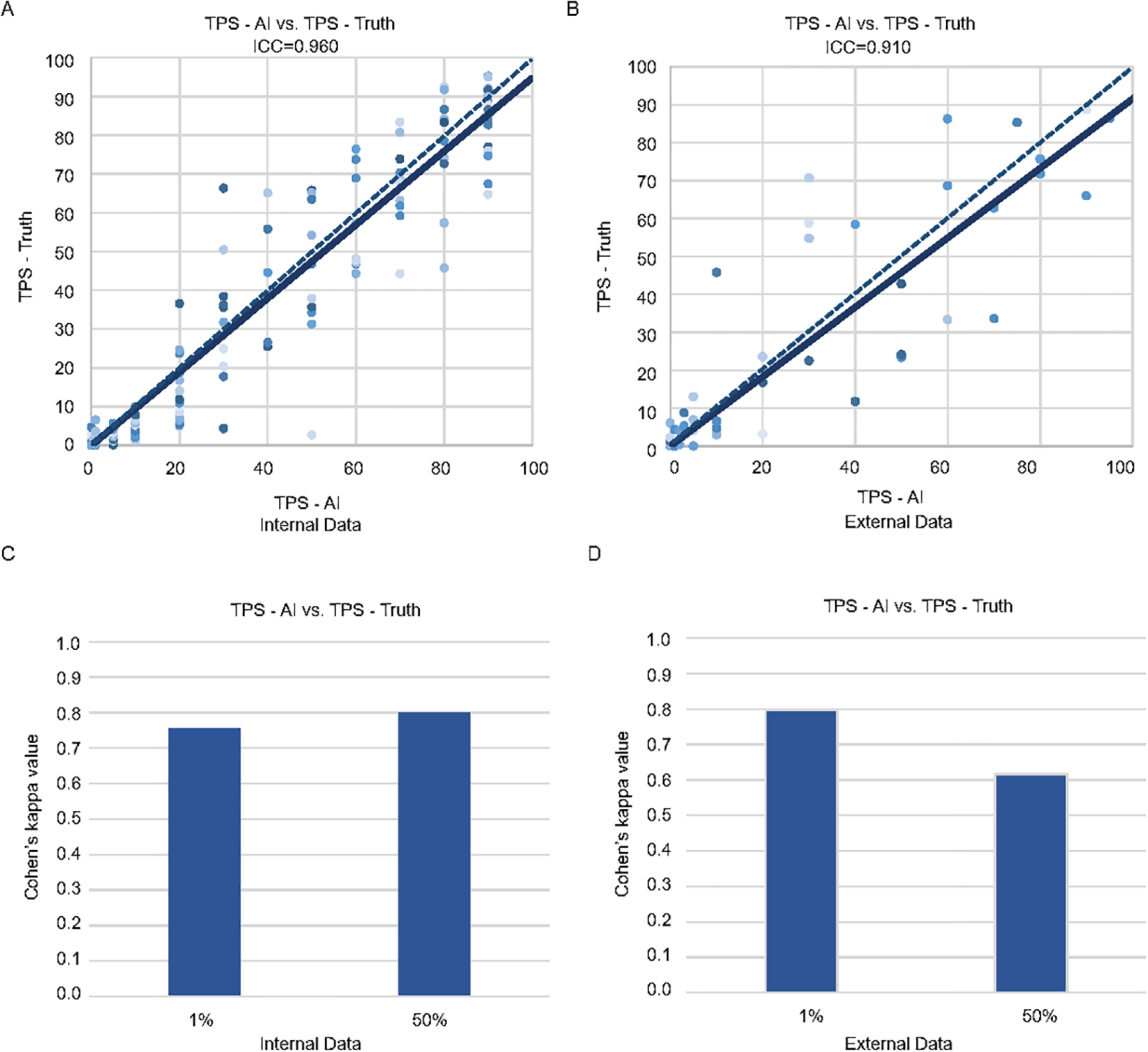
Consistency of the pathologists and MiLT in the internal and external test cohorts. Scatter plots of TPS-AI vs. TPS-Truth with intraclass correlation coefficient (ICC) in internal test cohort **(A)** and external test cohort **(B)**. Comparison of Cohen’s kappa values between AI and manual identification based on different cut-off values (1% and 50%) in internal test cohort **(C)** and external test cohort **(D)**.

For the MIL module training, the entire dataset is randomly partitioned into five equal sections. The first three sections are designated as the training set, while the fourth and fifth sections serve as the validation and test sets, respectively. The dataset records each WSI’s ID, the number of patches it contains, and the reference TPS values provided by pathologists, with 12 labels ranging from 0 to 0.9. Preprocessing steps such as RandomCrop, RandomHorizontalFlip, and RandomVerticalFlip are performed before model training. The structure of the prediction module (shown in **Figure 1**) uses ResNet18 as the base feature extraction model, with the final layer adjusted to output a feature vector of size num_features. The fully connected attention model applies an attention mechanism to these features, pools them using a distribution pooling filter, and prepares the representation for the final classification task.

To intuitively demonstrate the model’s predictive capabilities, the project developed a method for utilizing the trained MIL model for predicting and mapping probabilities across an entire slide. The slide is segmented into 256 x 256 pixel patches, with the coordinates of each patch recorded. These patches are organized into bags, each containing 200 patches, resulting in a total of 100 bags. The prediction value for each patch is assigned based on the overall predicted value of its corresponding bag. Additionally, the recalculation frequency of each patch is tracked, and the final prediction value for that patch is determined by averaging these values.

### 2.5 TPS calculation

TPS is calculated using the formula: (Number of PD-L1 positive tumor cells exhibiting weak to strong partial or complete membranous staining/Total number of tumor cells) × 100.


$$\text{TPS}=\;\frac{\text{PD}-\text{L}1\;\text{positive}\;\text{TCs}}{\;\text{viable}\;\text{TCs}}\times 100$$

To achieve a precise and accurate assessment of PD-L1 expression based on TPS, it is crucial to differentiate tumor cells from other cell types, including immune cells. Tumor cells are defined as PD-L1 positive whenever any partial or complete membranous staining is detected. A minimum of 100 viable tumor cells is required to determine the PD-L1 IHC positivity on a slide. Based on the levels of TPS expression, three subgroups have been established (1): No expression:<1% (2); Low expression: 1%-49% (3); High expression: >50%.

### 2.6 Statistical methods

A series of evaluation metrics were used to evaluate the performance of the developed model, including the ICC, Bland-Altman plots, kappa value, sensitivity, specificity, and confusion matrices at cut-off values of 1% and 50%.

The ICC is utilized to assess the consistency between the model’s TPS predictions and the reference TPS values provided by pathologists. Bland-Altman plots assess systematic bias and agreement limits. The mean difference reflects average prediction error. Upper/lower limits of agreement define the range within which 95% of differences lie. Similarly, the Kappa value is employed to determine the agreement between the model’s TPS predictions and the judgments made by medical professionals, correcting for random agreement and addressing biases and issues of precision between different assessment sources. The levels of agreement for Kappa can be classified into five categories: 0-0.2 as slight, 0.2-0.4 as fair, 0.4-0.6 as moderate, 0.6-0.8 as substantial, and 0.8-1.0 as almost perfect. The model’s predictive accuracy is evaluated using the Sensitivity and Specificity values at cut-off points of 1% and 50%. All assessment materials are generated using Python in the PyCharm IDE.

## 3 Results

### 3.1 Cohort clinical data description

As for the 439 lung cancer patients used for model building and internal testing, the histopathological specimens were randomly categorized at the level of individual patients into training and testing cohorts. **Table 1** summarizes the clinical and pathological characteristics of the patients in both the training and testing cohorts. The majority of the patients presented with primary tumors, and a minority exhibited metastatic disease. No statistically significant differences were observed between the training and testing groups (all p-values >0.05).

**Table 1:** Clinicopathological characteristics of patients.

Characteristics	Training cohort Case (n = 230)	Test cohort Case (n = 209)	*X* ^2^	*P*-value
Sex				
Male	138	129	0.136	0.712
Female	92	80		
Age (years)				
≤ 65	125	105	0.741	0.389
> 65	105	104		
Tumor type				
NSCLC	2	2	0.495	0.974
Lung Cancer	106	94		
LUSC	17	15		
LUAD	104	96		
Others	1	2		
Sampling methods				
Surgery	132	130	1.054	0.590
Percutaneous Biopsy	68	55		
Others	30	24		
Tumor origin				
Lung	203	194	3.202	0.202
Lymph	11	8		
Others	16	7		

NSCLC, non-small cell lung cancer; LUSC, lung squamous cell carcinoma; LUAD, lung adenocarcinoma.

### 3.2 Performance of model on tumor patch classification

**Figure 2:**
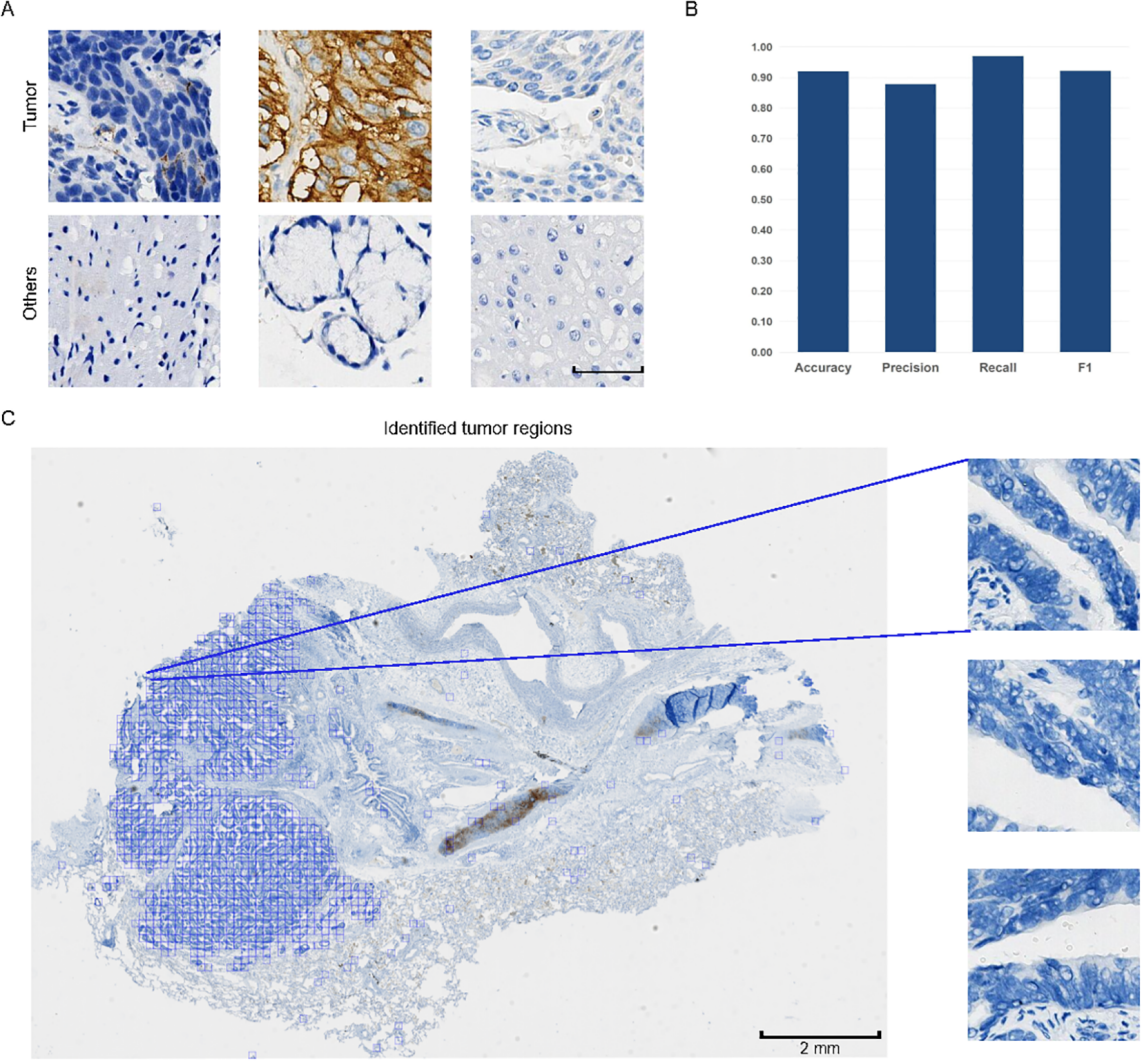
Examples of patches and performance of the classification model. **(A)** Typical patches of tumor areas and other regions. Scale bar: 50 μm. All patches are of the same size. **(B)** Evaluation metrics of the model’s performance on tumor patch classification, including Accuracy, Precision, Recall, and F1 score. The y-axis represents the metric values ranging from 0.00 to 1.00. **(C)** Pattern diagram of whole slide images (WSIs) divided into smaller patches of 256 x 256 pixels. Typical examples of tumor patches are magnified for better visualization. In the WSI, tumor patches are displayed, with the tumor regions marked in blue among all tumor patches.

TPS evaluation considers only PD-L1 positive tumor cells within tumor areas. To reduce the influence of regions without tumor cells on the development of MIL model, the entire process begins with using a classification model to differentiate tumor patches from non-tumor patches within WSIs. Our classification model segmented the WSIs into smaller patches of 256 x 256 pixels, successfully identifying and highlighting the tumor regions within WSIs (displayed by the blue box) (**Figures 2A–C**). These patches were subsequently saved for further processing. Notably, among the nearly 19,000 images constituting the test set (**Supplementary Figure S1**), the model accurately classified 92.09% of the data samples, demonstrating excellent performance in both recall rate and F1 score (**Figure 2B**). Based on high recognition rates, this model effectively distinguished tumor tissue from other tissue types.

### 3.3 Comparison of consistency between the model and pathologists

The classified tumor patches are then input into the MIL module, which produces the predicted TPS results (TPS-AI). To demonstrate the accuracy of MiLT, we evaluate the consistency between TPS-AI and TPS-Truth, and the ICC was used for continuous TPS values. Firstly, we evaluate the performance of MiLT on the held-out internal test set (n = 209), which was not seen by the model during training. Additionally, we introduced external data (n = 104) for validation to further verify the model’s generalization capability (**Figure 1B**, **Supplementary Table S1**). The results are illustrated in **Figure 1**. The ICC for the internal dataset was 0.960 (95% confidence interval [CI], 0.950-0.971), indicating an exceptionally high agreement between the model’s predictions and the pathologist-assigned scores (**Figure 1A**). The ICC for external data was 0.910 (95% CI, 0.870–0.938), although slightly lower than the internal dataset, the ICC for external data still reflected a substantial level of consistency, suggesting the robustness of the model when applied to diverse datasets beyond the original test set (**Figure 1B**).

The Bland-Altman analysis was used to evaluate systematic bias and agreement limits between AI-predicted TPS (TPS-AI) and TPS ground truth values (TPS-Truth). For the internal cohort (**Supplementary Figure S2**), the mean difference between TPS-AI and TPS-Truth was −2.01 (95% limits of agreement: 15.73 to −19.75), indicating a slight systematic underestimation by the AI model. The narrow spread of differences within these limits suggests moderate variability in prediction errors, consistent with the high ICC (0.960) observed in the internal validation. In contrast, the external cohort exhibited a smaller mean difference (−0.29) but wider limits of agreement (20.26 to −20.84), reflecting greater variability in prediction discrepancies (**Supplementary Figure S2**). This aligns with the marginally reduced ICC (0.910) for external data, likely attributable to cohort heterogeneity or divergent data distributions.

Furthermore, 1% and 50% cut-off values are specific to the 22C3 PD-L1 clone in non-small cell lung cancer (NSCLC) and in the current clinical are recommended as thresholds for patient stratification for immunotherapy. At these cutoffs, the kappa index is used to evaluate the consistency of the model in the internal and external datasets. At 1% cut-off value, the model demonstrated kappa values of 0.756 and 0.797 for the internal and external datasets, respectively, reflecting a high level of agreement in predictions (**Figures 1C, D**). When the cut-off value was set at 50%, the model achieved a kappa value of up to 0.799 in the internal testing (**Figure 1C**), while the external testing yielded a slightly lower kappa value of 0.617 (**Figure 1D**). Nonetheless, both values indicate a high level of consistency, further underscoring the robustness of the model. Overall, these findings validate the capability of MiLT to provide reliable assessments across diverse datasets.

### 3.4 Evaluation of MiLT effectiveness

**Figure 3:**
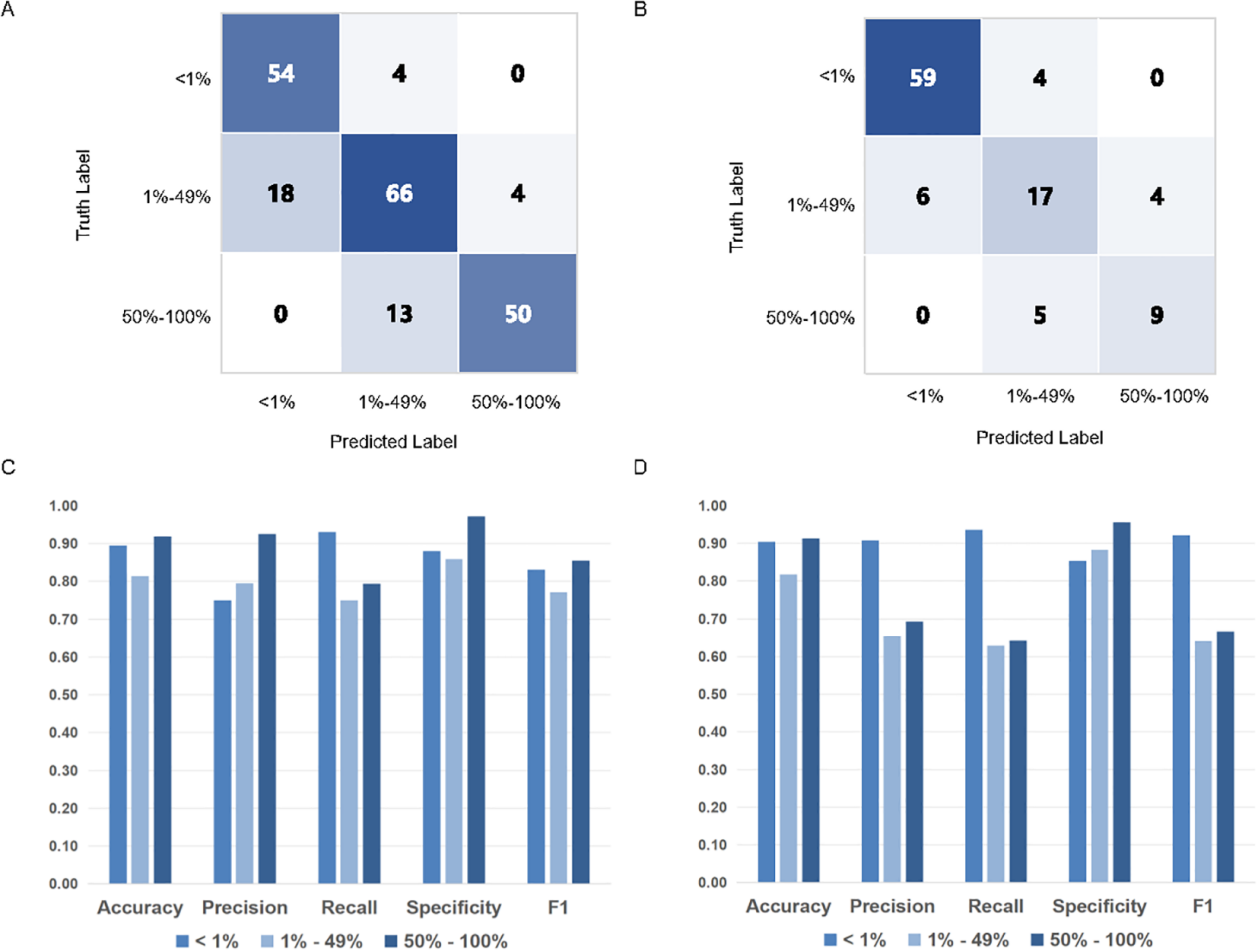
PD-L1 TPS assessments of MiLT and pathologists on different PD-L1 expression levels.The accuracy of TPS scores of AI based on confusion matrix analysis in internal test cohort **(A)** and external test cohort **(B)**. Comparison of histograms of DL model performance based on PD-L1 expression at different cut-off values (0% - 1% vs. 1% - 49% vs. 50% - 100%) in internal test cohort **(C)** and external cohort **(D)**.

To further evaluate MiLT effectiveness, confusion matrices were used to compare the accuracy of TPS scores predicted by AI. Next, using the results provided by experienced pathologists (TPS-Truth) as gold standard, the accuracy of TPS prediction by MiLT was separately evaluated use various assessment metrics (**Figures 3A–D**, **Supplementary Table S2**). In the internal test cohort, the model demonstrated excellent accuracy (0.813 to 0.919), recall (0.750 to 0.931), and specificity (0.860 to 0.973), indicating a strong overall classification capability for the samples (**Figure 3C**). The model performed exceptionally well in the<1% and 50%-100% ranges (**Figures 3A–D**). In the 1% to 49% range, the accuracy decreased to 0.813, though it remained at a relatively high level, with a slight increase in precision, suggesting an improvement in the model’s ability to identify false positives.

In the external dataset, the model maintained good accuracy and high specificity (**Figure 3D**). At the 50% cutoff, recall decreased, indicating a reduced ability of the model to identify cases with low PD-L1 expression, with a higher incidence of false negatives. The internal and external datasets further corroborated the model’s accuracy, although classification performance declined in the 1%-49% TPS range, it still remained at a relatively high level.

### 3.5 Visualization of model

**Figure 4:**
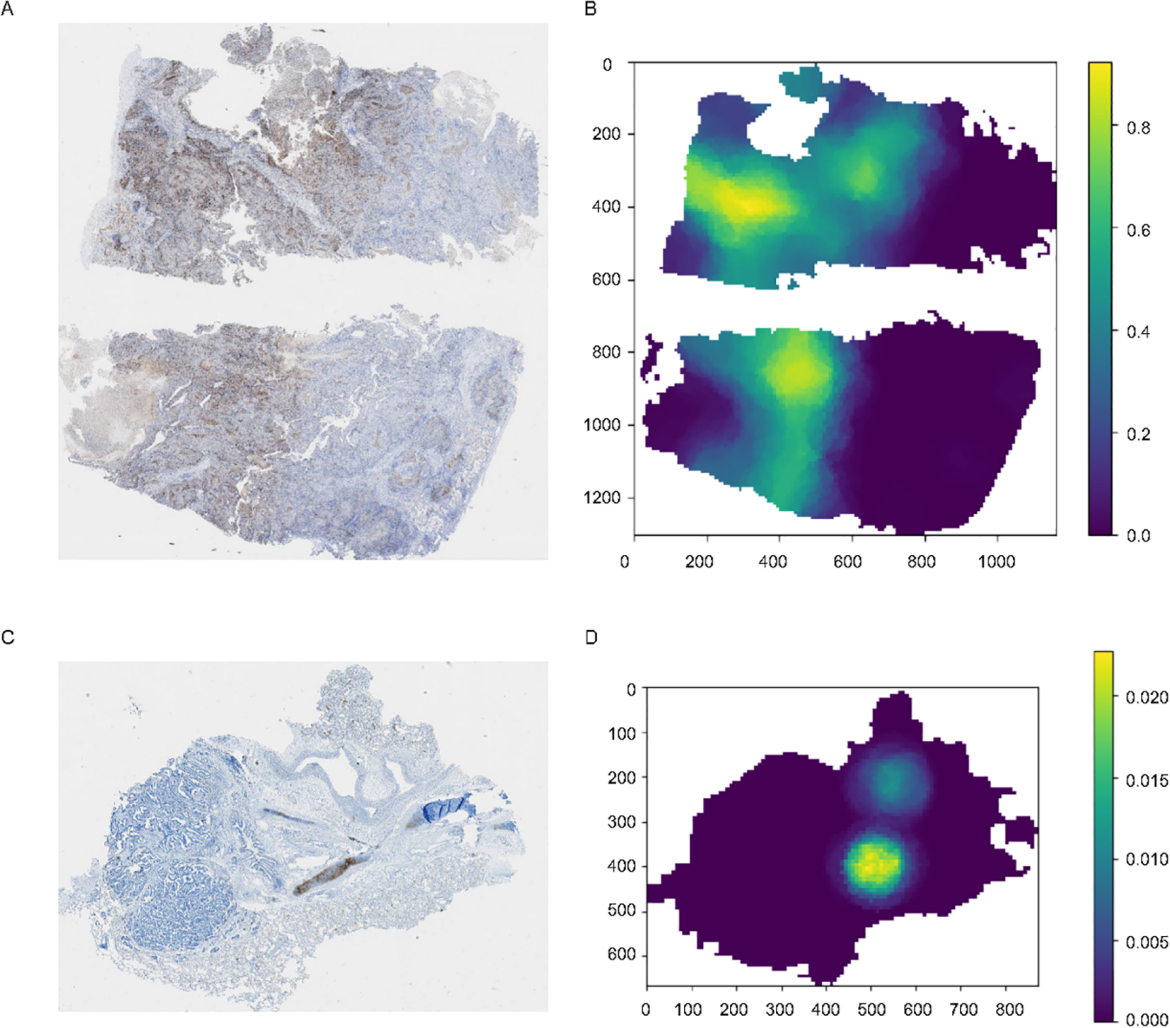
Original images and the corresponding heatmaps of model visualization. **(A, C)** The original images stained for PD-L1, with the brown-stained areas representing PD-L1 positive tissues. **(B, D)** Distribution heatmap for the entire image, set with different TPS thresholds.

Model visualization provides insights into the reasons and logic behind its predictions, and render the model more explainable to allow for monitoring of its performance once deployed. Additionally, visualization aids in debugging the model. Therefore, in this study, we employed heatmap visualization to predict the provided WSI using the model and generated a distribution heatmap for the entire image (**Figures 4A–D**). Specifically, we segmented the WSI into patches that were 256 x 256 pixels in size and organized these patches into bags of 10 x 10. Predictions were then made for all patches within each bag, and the average value was used to generate the heatmap for the entire image.

For images with varying staining intensities, such as **Figure 4C**, which represents a sample with a TPS< 1%, we can also adjust the threshold to observe regions of relatively strong expression throughout the entire WSI.

## 4 Discussion

The use of ICIs in immunotherapy is increasingly prevalent, and identifying populations that are likely to benefit from such therapies is central to determining effective treatment strategies (35). The TPS is a commonly used metric for screening effective patient populations and is typically interpreted manually by clinical pathologists (10, 36). However, there is considerable variability in these assessments, even among expert pathologists.

**Figure 5:**
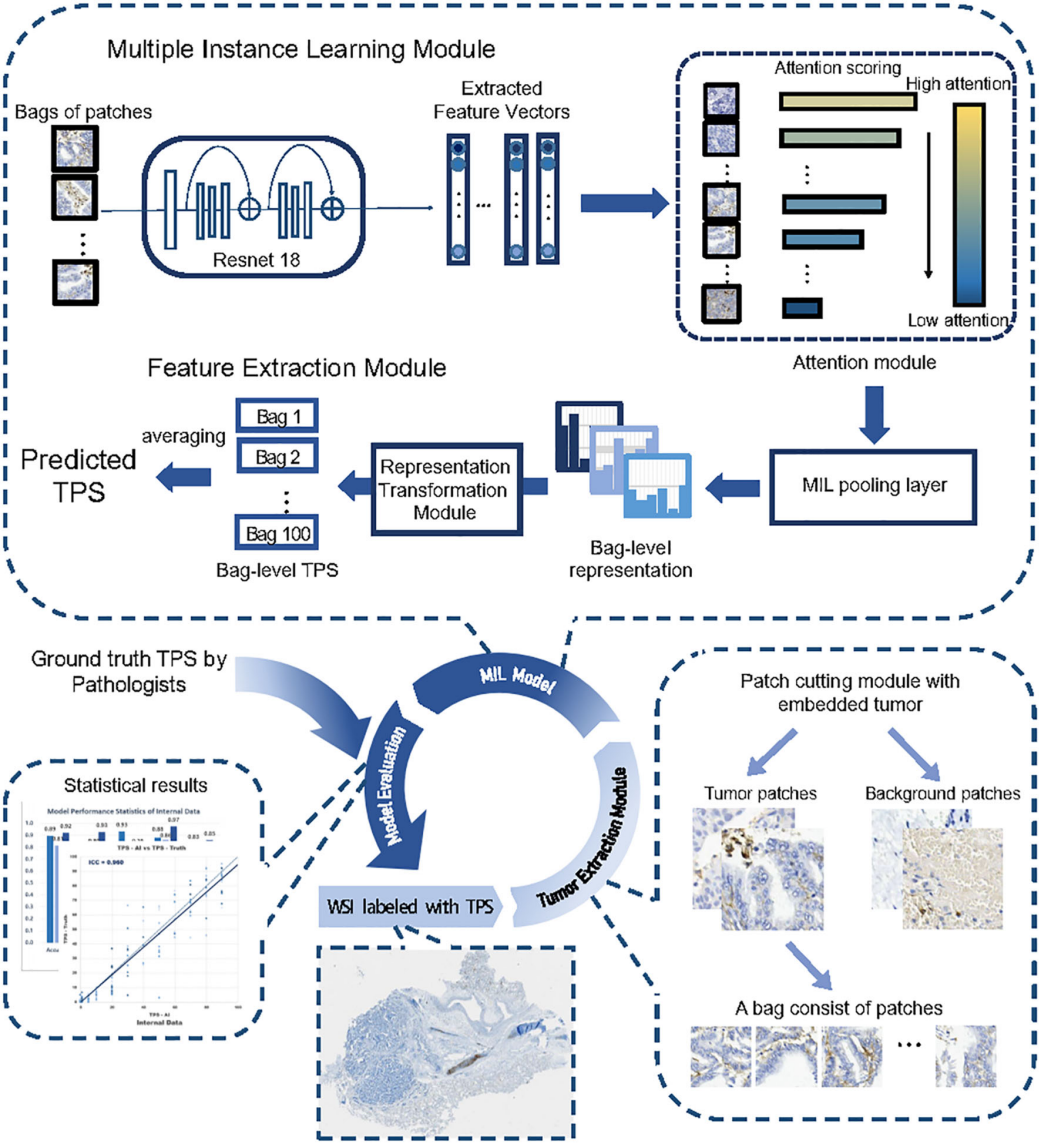
The entire workflow of the proposed deep learning framework. The entire workflow consisted of three parts, beginning by cropping the input WSI into patches and extracting tumor patches through the classification module, with patches randomly placed into bags. The core part was the MIL module, where the model took a bag of patches as input and predicted the sample’s TPS in its output. The feature extractor module extracted a feature vector for each patch within the bag. The attention module calculated attention scores based on the feature vectors and assigned weights to the patches. The MIL pooling filter summarized the extracted features into a bag-level representation by estimating the marginal feature distribution. Finally, the bag-level representation transformation module predicted the sample-level TPS. The TPS values inferred by multiple experienced pathologists were used as labels during training.

To assist pathologists in evaluating TPS, this study introduced MiLT, a DL-based framework taking advantage of MIL method to predict TPS in WSIs (**Figure 5**). MiLT can accurately identify tumor regions and predicts the proportion of PD-L1-positive cells within those regions, ultimately producing a TPS score. Nowadays, as MIL method use sample-level labels for training, which are weak labels and are easily collected from pathology reports, these methods are successfully used in various digital pathology (37), especially in the prediction of genetic alterations based on HE images (38, 39). However, most of these clinical scenarios use MIL to address a classification task, only a few researches are dedicated for the analysis of continuous variables and quantitative data (40). In this study, we treat PD-L1 score evaluation as multi-task problem using MIL method (33), and add a tumor extraction module before MIL process, demonstrating our pipeline with robust performance across both internal and external testing cohorts. In the internal test set, the predicted scores from MiLT show a high degree of consistency with those from pathologists, evidenced by a very high ICC (0.96) and strong kappa value (0.799). The model achieved an accuracy of 0.813, indicating excellent performance. We also selected an external cohort from other hospitals to serve as a validation set, thus reflecting an objective real-world clinical case environment. Even in external validation, our AI model exhibited reliable capabilities, achieving an accuracy rate of 81.7%. Our results show MiLT is a promising tool to aid clinical decision-making for cancer patients.

The application of MIL as a weakly supervised learning model alleviates the need for cell-level annotations, thereby requiring only WSI labels, enhancing current TPS assessment methods. Traditional prediction approaches predominantly rely on strong supervised learning, that requires extensive annotated data to maintain high accuracy, often necessitating cell-level labeling (24). However, sparse or biased data can lead to poor performance in strong supervised algorithms (41). Acquiring sufficient annotated data is particularly challenging when dealing with datasets from diverse institutions. From this perspective, our MIL approach mitigates dependence on abundant labeled data, allowing the model to generate reliable predictions based on weak annotations, thus serving as an effective assistant for clinical pathologists.

Time efficiency is also crucial for the practical application of predictive models. In classifying tumor and non-tumor patches, we utilize the classification method to detect the presence of tumor cells within the patches rather than pixel-level segmentation method, significantly accelerating identification speed. Furthermore, our MIL approach predicts the results for each bag rather than the entire WSI. The final TPS for the WSI is obtained by averaging predictions across all bags, greatly reducing prediction time and simplifying the process, with an average prediction time less than one minute per WSI, depending on image size.

This study has several limitations. Firstly, although our model has yielded satisfactory results, the dataset is relatively small. Gathering additional training data from multiple institutions would enhance the robustness of the AI model, and further clinical trials are also needed to validate the performance of the AI system in real-world settings. Secondly, the TPS scoring gold standard employed in this study is based on consensus readings by 2 or 3 experienced pathologists, which introduces a degree of subjectivity in the classification of heterogeneous cases. Thirdly, our study utilized only a single clonal kit (22C3), which may limit the generalizability of our findings in clinical application for other clonal kits. Future research should consider employing multiple clonal kits to ensure broader applicability and to better understand the potential variability in results due to differences in kit characteristics. Fourthly, AI models must be explainable to engender trust, the explainability of weakly supervised learning is inferior to strong supervised learning. Although MiLT provides heatmap of PD-L1 scores, with certain explainability, more explainability methods are need to explore (42). Additionally, the architecture of the model may not be optimal. On one hand, we speculate that improvements could be made by refining the bag sampling within the MIL model. On the other hand, in past few years, many studies have developed foundation models for digital pathology using hundreds of thousands or even millions of WIS to generate data representations, that can generalize well to diverse predictive tasks (43, 44). By replacing the feature extraction part of MIL module with a foundation model, the performance and robustness of MiLT may be further improved.

The introduction of MiLT has the potential to significantly impact current clinical practices in several ways. Firstly, by providing a standardized and automated method for TPS evaluation, MiLT can reduce the variability associated with manual assessments by different pathologists. This standardization is crucial for ensuring consistent treatment decisions across different clinical settings. Secondly, the time-efficient prediction capabilities of MiLT can streamline the workflow in pathology departments, allowing for faster and more efficient processing of WSIs. This efficiency can lead to quicker turnaround times for diagnostic reports, ultimately benefiting patient care.

Moreover, the adaptability of MiLT to various clinical standards makes it a versatile tool that can be integrated into existing pathology workflows with minimal disruption. The potential for integrating advanced AI in the evaluation of TPS opens avenues for further research and development in digital pathology. Future work should focus on exploring the broader applicability of MiLT in diverse clinical settings and addressing the limitations identified in this study. This includes gathering larger and more diverse datasets, employing multiple clonal kits, and refining the model architecture to improve performance and reliability.

In summary, MiLT serves as an effective tool for predicting TPS and demonstrates potential as a proof of concept for applying MIL methods in quantitative image analysis. The high technological performance and potential clinical benefits of MiLT warrant further investigation in prospective randomized clinical trials. Future research should aim to validate the model’s performance in real-world settings and explore its broader implications for clinical practice.

## Data Availability

The original contributions presented in the study are included in the article/**Supplementary Material**. Further inquiries can be directed to the corresponding authors.
