# Supplementary material for: A weakly supervised deep learning framework for automated PD-L1 expression analysis in lung cancer
Source: Front Immunol. 2025 Mar 31;16:1540087. doi: 10.3389/fimmu.2025.1540087 (PMC11994606; doi:10.3389/fimmu.2025.1540087)
Supplement: Supplementary file 1 [file DataSheet1.pdf]

Supplemental Material

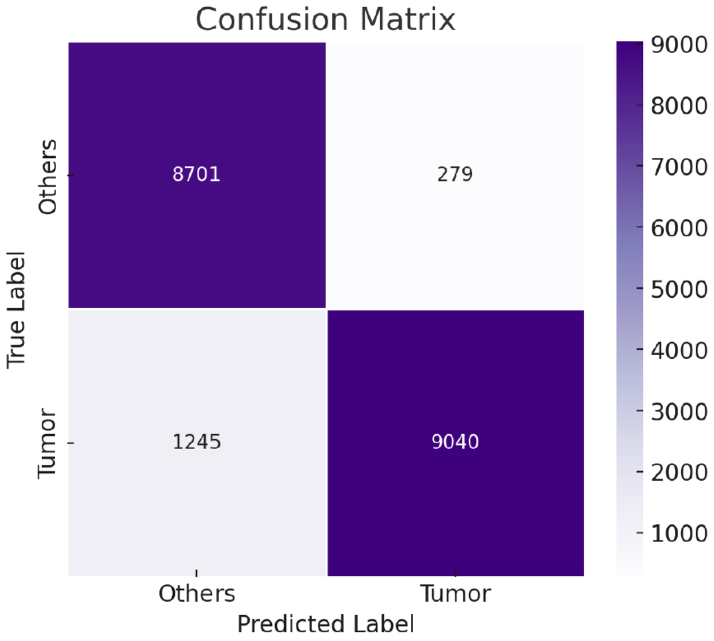

Figure S1. Confusion matrix for the performance analysis of tumor classification model.

The confusion matrix illustrates the accuracy of the tumor classification model in predicting tumor patches and other patches, including the number of false positives as well as the number of false negatives.

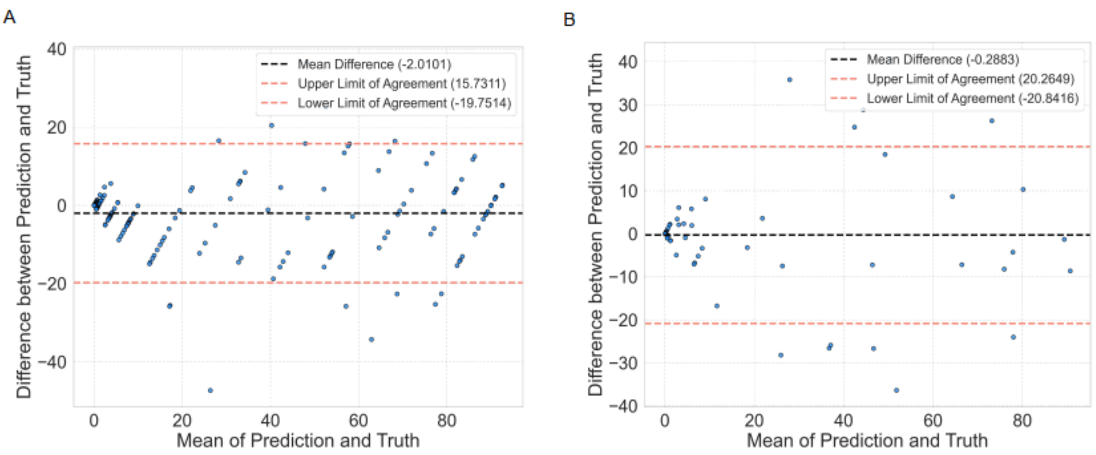

Figure S2. Consistency of the AI-predicted outcomes and the ground truth using Bland-Altman analysis.

Bland-Altman plots showing differences between TPS-AI and TPS-Truth in internal cohort (**A**) and external cohort (**B**). Mean difference (closer to 0 = less bias), agreement limits (narrower ranges = higher precision).

Table S1. The performance metrics and consistency among TPS-AI and TPS-Truth.

Statistics	Internal validation				External validation			
	**< 1%**	**1% - 49%**	**50% - 100%**	**Overall**	**< 1%**	**1% - 49%**	**50% - 100%**	**Overall**
**Accuracy**	0.8947 [0.8531, 0.9363]	0.8134 [0.7606, 0.8662]	0.9187 [0.8816, 0.9557]	0.8134 [0.7606, 0.8662]	0.9038 [0.8472, 0.9605]	0.8173 [0.7430, 0.8916]	0.9135 [0.8594, 0.9675]	0.8173 [0.7430, 0.8916]
**Precision**	0.7500 [0.6913, 0.8087]	0.7952 [0.7405, 0.8499]	0.9259 [0.8904, 0.9614]	0.8237 [0.7720, 0.8754]	0.9077 [0.8521, 0.9633]	0.6538 [0.5624, 0.7453]	0.6923 [0.6036, 0.7810]	0.7513 [0.6682, 0.8344]
**Recall**	0.9310 [0.8967, 0.9654]	0.7500 [0.6913, 0.8087]	0.7937 [0.7388, 0.8485]	0.8249 [0.7734, 0.8764]	0.9365 [0.8896, 0.9834]	0.6296 [0.5368, 0.7224]	0.6429 [0.5508, 0.7349]	0.7363 [0.6516, 0.8210]
**Specificity**	0.8808 [0.8194, 0.9233]	0.8595 [0.7865, 0.9104]	0.9726 [0.9317, 0.9893]	0.9043 [0.8644, 0.9442]	0.8537 [0.7156, 0.9312]	0.8831 [0.7925, 0.9373]	0.9556 [0.8912, 0.9826]	0.8974 [0.8391, 0.9558]
**F1**	0.8308 [0.7799, 0.8816]	0.7719 [0.7150, 0.8288]	0.8547 [0.8069, 0.9025]	0.8191 [0.7670, 0.8713]	0.9219 [0.8703, 0.9735]	0.6415 [0.5493, 0.7337]	0.6667 [0.5761, 0.7573]	0.7434 [0.6594, 0.8273]
**Kappa**	0.7557 [0.6519, 0.8431]	0.6143 [0.5071, 0.713]	0.7987 [0.7011, 0.8821]	0.7170 [0.6301, 0.7909]	0.7970 [0.6542, 0.9032]	0.5190 [0.3129, 0.7019]	0.6170 [0.3307, 0.8202]	0.6615 [0.5215, 0.7810]

Performance metrics of the artificial intelligence (AI) model for tumor proportion score (TPS) classification in internal and external validation cohorts, stratified by TPS categories (<1%, 1%-49%, 50%-100%) and overall results in internal and external datasets. Values represent point estimates with 95% confidence intervals in brackets.

Table S2. The ICC between AI and pathologists in tests.

Tests	ICC	95% CI	F-statistic	p-value
Internal cohort	0.960	0.950-0.971	49.389	1.33E-117
External cohort	0.910	0.870-0.938	21.348	1.35E-41

ICC, intraclass correlation coefficient; AI, artificial intelligence; CI, confidence interval.
